# Postoperative Pulmonary Hemodynamics and Systemic Inflammatory Response in Pediatric Patients Undergoing Surgery for Congenital Heart Defects

**DOI:** 10.1155/2022/3977585

**Published:** 2022-01-15

**Authors:** Maria Francilene S. Souza, Juliano G. Penha, Nair Y. Maeda, Filomena R. B. G. Galas, Kelly C. O. Abud, Eloisa S. Carvalho, Ana Maria Thomaz, Claudia R. P. Castro, Juliana Pereira, Antonio Augusto Lopes

**Affiliations:** ^1^Heart Institute (InCor), University of São Paulo School of Medicine, São Paulo, Brazil; ^2^Pró-Sangue Foundation, São Paulo, Brazil; ^3^Laboratory of Medical Investigation on Pathogenesis and Targeted Therapy in Onco-Immuno-Hematology (LIM-31), University of São Paulo, São Paulo, Brazil

## Abstract

There is scarce information about the relationships between postoperative pulmonary hemodynamics, inflammation, and outcomes in pediatric patients with congenital cardiac communications undergoing surgery. We prospectively studied 40 patients aged 11 (8–17) months (median with interquartile range) with a preoperative mean pulmonary arterial pressure of 48 (34–54) mmHg who were considered to be at risk for postoperative pulmonary hypertension. The immediate postoperative pulmonary/systemic mean arterial pressure ratio (PAP/SAP_IPO_, mean of first 4 values obtained in the intensive care unit, readings at 2-hour intervals) was correlated directly with PAP/SAP registered in the surgical room just after cardiopulmonary bypass (*r* = 0.68, *p* < 0.001). For the entire cohort, circulating levels of 15 inflammatory markers changed after surgery. Compared with patients with PAP/SAP_IPO_ ≤ 0.40 (*n* = 22), those above this level (*n* = 18) had increased pre- and postoperative serum levels of granulocyte colony-stimulating factor (*p* = 0.040), interleukin-1 receptor antagonist (*p* = 0.020), interleukin-6 (*p* = 0.003), and interleukin-21 (*p* = 0.047) (panel for 36 human cytokines) and increased mean platelet volume (*p* = 0.018). Using logistic regression analysis, a PAP/SAP_IPO_ > 0.40 and a heightened immediate postoperative serum level of macrophage migration inhibitory factor (quartile analysis) were shown to be predictive of significant postoperative cardiopulmonary events (respective hazard ratios with 95% CIs, 5.07 (1.10–23.45), and 3.29 (1.38–7.88)). Thus, the early postoperative behavior of the pulmonary circulation and systemic inflammatory response are closely related and can be used to predict outcomes in this population.

## 1. Introduction

Pulmonary hypertension is an important factor influencing the outcomes following pediatric cardiac surgery. Young patients with unrestrictive congenital cardiac communications have altered pulmonary hemodynamics associated with functional and structural changes in the pulmonary vascular bed. These abnormalities pose a risk for immediate postoperative clinical instability and residual pulmonary hypertension long after the repair of cardiac lesions, particularly in individuals not treated in a timely fashion (early in life). A number of perioperative factors contribute to postoperative systemic and pulmonary vascular tone instability, leading to hemodynamic disturbances. Cardiopulmonary bypass (CPB) with altered flow conditions, tissue ischemia and reperfusion, hypothermia, and the use of blood products or protamine elicit a systemic inflammatory response [1]. Superimposed on preexisting endothelial cell dysfunction, the inflammatory reaction elicits an increased propensity to spasm in the pulmonary circulation [2]. Cell activation (of endothelial cells, leukocytes, and platelets), which involves the expression of cytokines and adhesion molecules and the production and release of reactive oxygen species, arachidonic acid derivatives, and proteolytic enzymes are events that take place during and after CPB, leading to vasomotor disturbances and multiorgan dysfunction [3–6]. Heightened circulating levels of endothelins and changes in the L-arginine-nitric oxide-cGMP pathway further contribute to pulmonary vascular reactivity [2]. The pulmonary vascular bed responds dramatically to changes in pH. Postoperatively, alveolar edema, ventilation-perfusion mismatch, and bronchoconstriction contribute to hypoxia, which may either trigger or exacerbate pulmonary vasoconstriction [2, 7].

In more privileged communities, the contemporary incidence of postoperative pulmonary hypertensive crises is probably less than 1% [2]. There have been estimates ranging between 2% and 5%, with higher percentages in specific subsets of patients, such as those with Down syndrome (~10%) and those with atrioventricular septal defect (14%) [8]. The lack of firm diagnostic criteria makes it difficult to have precise estimates of the incidence of crises. Very likely, the estimates of ~1% refer to episodes of severe and sustained pulmonary artery pressure rise with hypoxemia and systemic hypotension, with still unacceptably high mortality rates of 20% to 30% [2, 9]. A pulmonary-to-systemic arterial pressure ratio above 0.75 with a decrease in systemic arterial pressure of 20% or more and a systemic oxygen saturation below 90% have been used as criteria to define pulmonary hypertensive crises [7]. In some reports, however, crises have been defined as a rise in pulmonary arterial pressure to or above the systemic arterial pressure level, with no further specifications about the duration of episodes or the occurrence of concomitant disturbances [10]. Strict definitions may be problematic in that they do not provide a realistic view of hemodynamic abnormalities that occur in this population. Transient elevations in pulmonary arterial pressure, even to the systemic level, that are promptly reversed by manual ventilation, sedation, and muscle relaxants probably do not have an impact on short-term outcomes. On the other hand, some episodes of pulmonary artery pressure rise, even if not severe enough to cause significant hemodynamic impairment, last longer, or occur in clusters, requiring substantial changes in therapeutic measures. In the presence of advanced right ventricular dysfunction, low cardiac output with systemic hypotension may be the dominant scenario rather than marked elevations of pulmonary arterial pressure [11]. While many of these instabilities are not necessarily associated with high mortality rates, they are major contributors to the need for prolonged mechanical ventilation and the length of stay in the hospital.

This study was planned to investigate the relationships between hemodynamics, postoperative clinical events, and inflammatory profiles in a prospective cohort of young pediatric patients undergoing surgery for congenital heart disease who were considered to be at risk for postoperative pulmonary hypertension. This assumption of risk was based on the fact that they represented the population of a tertiary referral center with a high prevalence of Down syndrome and had unrestrictive cardiac communications with elevated pulmonary artery pressure, as suggested by preoperative echocardiographic evaluation. Postoperative hemodynamics was evaluated by continuously registering pulmonary and systemic arterial pressures for 2.5 days. We adopted a more flexible definition of postoperative cardiopulmonary events by including episodes that would not be interpreted as typical pulmonary hypertensive crises. However, we disregarded transient and isolated elevations in pulmonary arterial pressure. Inflammatory profiles were characterized by measuring the circulating levels of several inflammatory markers preoperatively and in the early postoperative period.

## 2. Methods

### 2.1. Study Population

This single-center study was performed at the Heart Institute (InCor), University of São Paulo School of Medicine, São Paulo, Brazil. Patients were consecutively enrolled from November 2016 to October 2018 based on the following criteria: age 1 month to 3 years; presence of unrestrictive cardiac communications, i.e., with a diameter of the posttricuspid communication greater than 50% of the aortic annulus diameter on transthoracic echocardiography; absence of pulmonary stenosis; hearts with biventricular physiology; clinical features suggestive of at least moderately elevated pulmonary arterial pressure; absence of extracardiac syndromes other than Down syndrome; and absence of any signs of ongoing or recent inflammatory or infectious diseases. In this study, patients were assigned to cardiac surgery essentially on the basis of noninvasive elevation. Patients with peripheral oxygen saturation lower than 85% and significant right-to-left shunting across the cardiac septal defects, suggesting the presence of advanced pulmonary vasculopathy, were not included. Healthy pediatric subjects with an age as close as possible to the age range of the patients were recruited from the same geographic area and entered the study as controls for the laboratory (biochemical) tests. This group was also made up of individuals with Down syndrome. Informed consent signed by family members was necessary for inclusion in all instances. The study protocol was approved by the Institutional Scientific and Ethics Committee (CEP n^o^. 2.068.696).

### 2.2. Perioperative Protocols and Hemodynamic Parameters

Some patients had clinical and radiographic signs of pulmonary overcirculation, often associated with a pulmonary/systemic blood flow ratio greater than 2.5 and peripheral oxygen saturation > 93%. Subjects with no signs of congestive heart failure or failure to thrive, with a pulmonary/systemic blood flow ratio of <2.0 and peripheral oxygen saturation ≤ 93%, were considered to have higher levels of pulmonary vascular resistance, so they required special attention perioperatively. Transthoracic echocardiography was performed preoperatively to assess anatomic and functional parameters. Systolic pulmonary artery pressure was obtained from the tricuspid regurgitation gradient. Mean pulmonary artery pressure was calculated as (0.61 × systolic pressure) + 2 mmHg [12]. The pulmonary/systemic blood flow ratio and pulmonary venous flow were estimated based on flow parameters in the right and left ventricular outflow tracts and the velocity-time integral of blood flow in the pulmonary veins. Right ventricular systolic function was assessed by measuring the tricuspid annular plane systolic excursion (TAPSE).

Postoperatively, patients were kept under analgesia/sedation according to institutional protocols. In most instances, fentanyl, midazolam, and ketamine were used singly or in combination. Milrinone, epinephrine, and norepinephrine were used as inotropic/vasoactive agents. Inhaled nitric oxide (10–20 ppm) was started in the operating room immediately after CPB termination and was maintained throughout the entire period of mechanical ventilation. Pulmonary and systemic arterial pressures were measured directly in the operating room and by using indwelling catheters in the postoperative care unit. Intraoperatively, pressures were recorded before and after CPB. Postoperatively, readings were taken at 2-hour intervals (12 times a day) for 2.5 days. Pulmonary and systemic arterial pressures were computed, and pressure curves were constructed. The pulmonary/systemic mean arterial pressure ratio (PAP/SAP) was calculated. Immediate postoperative pulmonary hemodynamics was defined as the mean of the first 4 values (PAP/SAP_IPO_), corresponding to the first 6 hours of postoperative care. This parameter was used to characterize hemodynamic groups.

### 2.3. Postoperative Cardiopulmonary Events (Study Outcome)

After surgery, we computed interrelated hemodynamic and respiratory disturbances of varying severity, from elevated pulmonary arterial pressure (>75% of systemic arterial pressure level) with a decline in systemic pressure (≥20%) and oxygen desaturation (<90%) to more severe hypoxemia and hypotension requiring manual ventilation and eventually cardiorespiratory resuscitation. In some instances, pulmonary arterial pressure was not seriously elevated at the onset of clinical instability (for example, 50%-75% of systemic pressure level). Rather, systemic hypotension was the dominant feature requiring changes in the doses of vasoactive-inotropic drugs. In the study, these disturbances are referred to as significant cardiopulmonary events (SCAPEs). Some episodes were prolonged (i.e., not promptly responsive to sedation and manual ventilation) and/or recurrent. Importantly, transient elevations of pulmonary pressure, even to suprasystemic levels, that were rapidly reversed by manual ventilation were not categorized as SCAPES. Interpretations were made independently by 3 physicians (AAL, AMT, and FRBGG). Patients whose systemic arterial pressure stabilized upon recovery from SCAPEs were started on sildenafil (four daily doses of 0.25 mg/kg via nasoenteral tube) and kept on it until nitric oxide discontinuation and weaning from mechanical ventilation or for the rest of their hospital stay.

### 2.4. Microcirculatory Parameters and Inflammatory Markers

Hemodynamics and microcirculatory status were monitored via pulmonary and systemic pressure curves, mixed venous oxygen saturation, and serum lactate, particularly on the first 2 to 3 days after surgery. The amount of cardiovascular support required postoperatively was computed using the vasoactive-inotropic score [13]. To investigate the relationships between the patterns of pressure curves, SCAPEs, and inflammatory profiles, inflammatory markers were analyzed preoperatively and 4 hours and 24 hours after CPB. We analyzed serum levels of 36 specific inflammatory mediators: complement component 5/5a (C5/C5a); CD40 ligand (CD40L); granulocyte colony-stimulating factor (G-CSF); granulocyte-macrophage colony-stimulating factor (GM-CSF); growth-regulated oncogene alpha (GRO*α*); human CC chemokine I-309 (I-309); intercellular adhesion molecule-1 (ICAM-1); interferon gamma (IFN-*γ*); interleukins (IL) 1 alpha, 1 beta, 2, 4, 5, 6, 8, 10, 12, 13, 16, 17, 17E, 18, 21, 27, and 32 alpha; interleukin-1 receptor antagonist (IL-1RA); interferon gamma-induced protein-10 (IP-10); interferon-inducible T cell alpha chemoattractant (I-TAC); monocyte chemoattractant protein-1 (MCP-1); macrophage migration inhibitory factor (MIF); macrophage inflammatory protein-1 alpha/beta (MIP-1*α*/*β*); regulated on activation, normal T cell expressed and secreted (RANTES); plasminogen activator inhibitor-1 (Serpin E1); stromal cell-derived factor-1 (SDF-1); tumor necrosis factor alpha (TNF-*α*); and soluble triggering receptor expressed on myeloid cell-1 (sTREM-1). Proteins were analyzed by immunoblotting using a human cytokine array (R&D Systems, Minneapolis, MN, USA). Samples were processed in duplicate, and proteins were detected semiquantitatively by chemiluminescence. The results are expressed as the average signal of each pair of duplicate spots (units of pixel intensity, upi). For all patients, preoperative and postoperative samples were run in the same assay. Samples from pediatric controls were run in parallel. The control group was within the same age range of patients and included individuals with Down syndrome as well.

### 2.5. Data Obtainment

In this study, preoperative and postoperative assessments (responsible authors, MFSS, JGP, FRBGG, KCOA, ESC, AMT, and CRPC) and laboratory analyses (NYM) were carried out in a blinded fashion.

### 2.6. Statistical Analysis

Unless otherwise specified, numeric variables are presented as medians with interquartile ranges. Categorical variables are presented as number of cases and percentages, and differences between groups were tested using the Chi-square family of tests. The Wilcoxon test, Friedman's test, and Pearson's coefficient of correlation were used to test for differences and associations within subjects. Inferential statistics were used to analyze postoperative hemodynamics, biological markers, and microcirculatory parameters in hemodynamic groups, as well as potential predictors of outcome. For this purpose, the majority of variables were analyzed after Box-Cox transformation with further testing for the normality of their distributions. The general linear model was used to test for differences between groups at baseline and postoperatively. The general linear model for repeated measures was used to analyze arterial pressure and oxygen saturation curves postoperatively and differences between hemodynamic groups regarding data obtained at baseline and 4 hours and 24 hours after surgery. Logistic regression (univariate and multivariate analyses) was used to identify possible predictors of postoperative cardiopulmonary events. Receiver operating characteristic (ROC) curves were constructed, and sensitivity and specificity levels associated with cutoff values were determined. In all tests, 0.05 was set as the significance level. Statistical analysis was performed using the SPSS statistical software, version 26 (IBM, Armonk, NY, USA).

## 3. Results

### 3.1. Descriptive Analysis

Preoperative and intraoperative data of 40 patients who underwent surgery under CPB are listed in Table 1. There was a high proportion of patients with Down syndrome presenting with a complete atrioventricular septal defect. Elevation of pulmonary vascular resistance was suggested by increased pulmonary arterial pressure in the presence of relatively restricted pulmonary blood flow considering the size of the cardiac communications. Preoperative right ventricular systolic function (TAPSE) was roughly normal in most subjects. Pulmonary hypertension was further confirmed by an intraoperative pulmonary/systemic mean arterial pressure ratio of 0.73 (0.56–0.86) (median with interquartile range). Pulmonary arterial pressure decreased significantly after CPB in comparison with pre-CPB level, but it remained at ~40% of the systemic arterial pressure level (Table 1). Despite the use of inhaled nitric oxide, which was started in the operating room and maintained throughout the period of mechanical ventilation, 11 patients had SCAPEs of varying severity postoperatively. SCAPEs contributed to prolonged mechanical ventilation (*p* < 0.001) but not mortality. Other factors associated with prolonged mechanical ventilation (>7 days in 22 patients) were delayed sternal closure, respiratory disturbances, infection, arrhythmias, seizures, and other neurological manifestations. One patient died of septicemia. Another patient died of rapid-onset systemic hypotension followed by bradycardia unresponsive to vasopressin and other life-supporting interventions. There was no need for reoperation in the present cohort.

### 3.2. Hemodynamic Groups

**Figure 1:**
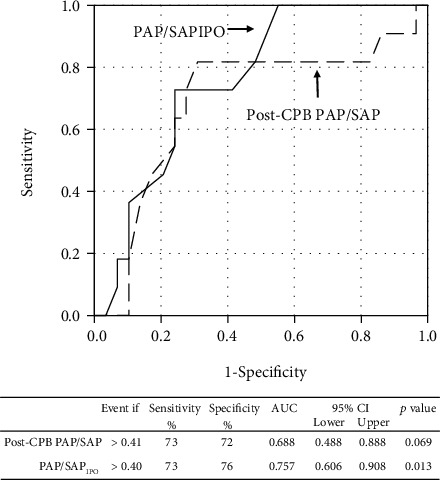
Receiver operating characteristic curves and cutoff values for pulmonary/systemic mean arterial pressure ratio (PAP/SAP) obtained just after cardiopulmonary bypass (post-CPB) and during the immediate postoperative period (IPO) in the prediction of *significant cardiopulmonary events* during the intensive care unit stay.

**Figure 2:**
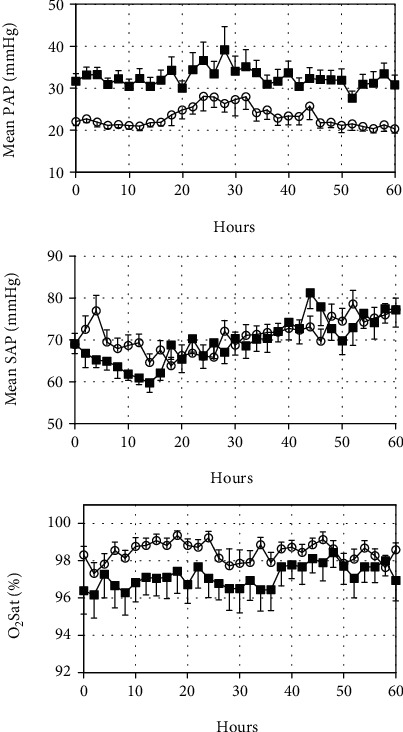
Pulmonary arterial pressure (PAP), systemic arterial pressure (SAP), and peripheral oxygen saturation (O_2_ Sat) measured in the postoperative care unit with readings at 2-hour intervals (12 times within a day) for 2.5 consecutive days. Data are presented as mean with SE. Circles and squares represent patients with PAP/SAP_IPO_ (immediate postoperative period) ≤ 0.40 (*n* = 22) and >0.40 (*n* = 18), respectively. Data were analyzed using the general linear model for repeated measures. There was a significant between-group difference for mean PAP (*p* < 0.001) but not for mean SAP (*p* = 0.439) or O_2_ Sat (0.113).

While analyzing postoperative hemodynamics, we observed that PAP/SAP_IPO_ (0.38 (0.32–0.46), range 0.19 to 0.67) was directly correlated with the PAP/SAP registered in the surgical room just after CPB discontinuation (*r* = 0.68, *p* < 0.001). PAP/SAP_IPO_ was even better than post-CPB PAP/SAP at predicting SCAPEs (Figure 1). The cutoff value of 0.40 obtained for PAP/SAP_IPO_ was very close to the median value of 0.38. Based on these observations, we decided to use PAP/SAP_IPO_ (≤0.40 versus >0.40) to categorize postoperative pulmonary hemodynamics and analyze factors that might explain differences in hemodynamic behavior. Compared with patients with PAP/SAP_IPO_ ≤ 0.40 (*n* = 22), those with PAP/SAP_IPO_ > 0.40 (*n* = 18) had greater pulmonary arterial pressure for the entire period of hemodynamic monitoring (Figure 2). Systemic arterial pressure did not differ between the groups, while peripheral oxygen saturation tended (nonsignificantly) to be lower in subjects with PAP/SAP_IPO_ > 0.40 during the first 36 hours. Preoperative and intraoperative data in hemodynamic groups are shown in Table 2. In the group of patients with PAP/SAP_IPO_ > 0.40, there was a greater proportion of individuals with an atrioventricular septal defect. A longer time was required to repair their cardiac lesions (CPB duration) in comparison with patients with PAP/SAP_IPO_ ≤ 0.40. Besides, in patients with PAP/SAP_IPO_ > 0.40, preoperative oxygen saturation was slightly but significantly lower. Notably, atrioventricular septal defects were present exclusively in subjects with Down syndrome. CPB time was 144 (108–157) minutes and 103 (90–125) minutes in syndromic and nonsyndromic individuals, respectively (*p* = 0.009). Also, Down syndrome patients had lower preoperative peripheral oxygen saturation compared with nonsyndromic subjects (96% (93%-98%) and 98% (96%-99%), respectively, *p* = 0.006).

### 3.3. Hemodynamics and Inflammatory Response

Preoperative and postoperative hematological parameters are shown in Table 3. Postoperatively, there was a decrease in lymphocyte count, while circulating monocytes and neutrophils and the neutrophil-to-lymphocyte ratio increased, compared to baseline. There was a decline in platelet count (no platelet transfusions we required) while the mean platelet volume and plasma C-reactive protein increased. The preoperative levels of the specific inflammatory proteins investigated in this study did not differ significantly between patients and controls, except for the chemokine RANTES, which was higher in patients (Table 4). Inflammatory markers were further analyzed for possible differences within subjects and between hemodynamic groups as defined. The levels of several markers changed significantly within subjects (measurements performed before surgery versus 4 hours and 24 hours after CPB termination) in both groups (Table 5). For example, IL-1RA was markedly elevated 4 hours after CPB compared to baseline, and it later declined. Similar increases were observed for other proteins, whereas the levels of CD40L, GRO*α*, Serpin E1, RANTES, and SDF-1 decreased after surgery. Serum CD40L correlated positively with platelet count before surgery (*r* = 0.41, *p* = 0.012) and 4 hours and 24 hours following CPB (*r* = 0.35, *p* = 0.035 and *r* = 0.41, *p* = 0.011, respectively). In addition, there was a negative correlation between serum RANTES and vasoactive-inotropic score (*r* = −0.57, *p* < 0.001 and *r* = −0.32, *p* = 0.043, respectively, 4 hours and 24 hours after CPB). Differences between the 2 groups were observed in 5 markers, namely, G-CSF, IL-1RA, IL-6, IL-21, and mean platelet volume, all of them higher in patients with PAP/SAP_IPO_ > 0.40 (Table 5).

### 3.4. Outcome Prediction

By univariate and multivariate logistic regression analyses, variables were tested for their association with SCAPEs.  Table 6 shows the variables for which a *p* value of <0.10 was obtained in univariate analysis. Except for the neutrophil-to-lymphocyte ratio, which appeared in the regression analysis with a negative coefficient, the variables shown in the table were positively correlated with the occurrence of SCAPEs. Early postoperative hemodynamics (PAP/SAP_IPO_) and IL-16, MIF, and neutrophil-to-lymphocyte ratio measured 4 hours after CPB were associated with a *p* < 0.05. However, using the forward LR procedure for variable inclusion, only PAP/SAP_IPO_ and MIF remained in the predictive model. The hazard ratio associated with a PAP/SAP_IPO_ > 0.40 was 5.07 (95% CI 1.10–23.45, *p* = 0.038); for post-CPB MIF analyzed as quartiles, it was 3.29 (95% CI 1.38–7.88, *p* < 0.001). Variables not significantly correlated with the occurrence of SCAPEs were patient age and presence/absence of Down syndrome; preoperative oxygen saturation, pulmonary/systemic blood flow ratio, and right ventricular systolic function (TAPSE); pre- and post-CPB pulmonary/systemic mean arterial pressure ratio and CPB time; postoperative vasoactive inotropic score, serum lactate, and mixed venous oxygen saturation; and serum levels of inflammatory markers (pre- and postoperative) not shown in Table 6.

## 4. Discussion

In this study, we analyzed the behavior of pulmonary and systemic arterial pressure curves and the occurrence of SCAPEs and investigated how they might be related to systemic inflammation, which we assessed by measuring circulating levels of inflammatory markers pre- and postoperatively. Pulmonary hemodynamic patterns were identified early after surgery and correlated with the levels of 5 inflammatory markers. SCAPEs were related to immediate postoperative hemodynamics and serum level of MIF protein and constituted one of the causes of prolonged mechanical ventilation. The variable PAP/SAP_IPO_ was characterized as a new and potentially useful parameter. We looked at PAP/SAP_IPO_ prior to analyzing the entire pressure curves because during the first 6 hours of postoperative care, patients were still deeply sedated on mechanical ventilation and relatively stabilized in terms of post-CPB physiology. Our findings suggest that the link between Down syndrome and pulmonary hypertension in the setting of congenital heart disease is constituted by features that are present in some but not all syndromic patients (e.g., atrioventricular septal defect and systemic oxygen desaturation) and becomes more evident under certain circumstances (longer surgical times). In some patients with Down syndrome, particularly those with atrioventricular septal defect, long perfusion times probably contributed to the upward shift of pulmonary pressure curve after surgery, which was clearly associated with systemic inflammation. Patients with this profile seemed to be at a higher risk of having a difficult postoperative course.

The overall postoperative inflammatory reaction was characterized by changes in the levels of several inflammatory markers, as shown in Table 4. The levels of most markers increased from baseline, while the levels of 5 proteins actually decreased after surgery: CD40L, RANTES, GRO*α*, Serpin E1, and SDF-1. We were probably unable to detect higher levels for GRO*α*, Serpin E1, and SDF-1 because peak concentrations of these proteins generally occur just after or even during CPB [17–19]. The decrease in CD40L was probably a result of the decrease in the number of circulating platelets after surgery. Platelets are a major source of CD40L [20] in addition to its expression by activated CD4+ T cells [21]. In our patients, serum levels of CD40L correlated positively with platelet counts pre- and postoperatively. The chemokine RANTES was increased in patients versus controls preoperatively, confirming previous observations [22]. RANTES declined postoperatively, probably as a result of the use of inotropic and vasopressor agents. RANTES is produced by human vascular endothelial cells under proinflammatory conditions [23], as well as being expressed by activated T cells and platelets [24]. In pediatric cardiac surgery, circulating RANTES has been shown to decrease, with lower levels being associated with longer surgical times, inotropic support, and reoperation [25]. In our study, postoperative RANTES levels correlated negatively with the vasoactive-inotropic score. Furthermore, CPB can cause a marked reduction in T lymphocytes [26] and the concentrations of macrophage-recruiting chemokines, including MIP-1*β* and RANTES. The recruitment and activation of neutrophil granulocytes are associated with multiorgan injury [27]. In the present study, CPB was followed by a decrease in lymphocyte count with a marked rise in the neutrophil-to-lymphocyte ratio.

Of the inflammatory markers that were analyzed in the study, IL-6, IL-1RA, G-CSF, IL-21, and mean platelet volume were closely correlated with early postoperative hemodynamic patterns, while higher MIF concentrations 4 hours post-CPB were associated with the occurrence of SCAPEs. In general, IL-6 and IL-1RA are proinflammatory and anti-inflammatory mediators, respectively. G-CSF and IL-21 have effects that characterize them as anti-inflammatory cytokines [28, 29]. In the pathophysiological context of this study, the increase in platelet volume may be interpreted as a proinflammatory event [30]. In pediatric cardiac surgery, increased plasma and tissue concentrations of proinflammatory cytokines such as IL-6 and IL-8 are balanced by a phased plasma (IL-10 followed by IL-1RA) and tissue (IL-10) anti-inflammatory responses [31, 32]. The ~10-fold increase in serum IL-1RA that we observed postoperatively, in the absence of any significant increase in the level of IL-1*α* or IL-1*β* (not shown), was not surprising. Because of the spare receptor effect, large quantities (~100-fold) of IL-1RA are required to functionally inhibit the biological effects of negligible amounts of IL-1 [33]. Thus, in the study, the increase in IL-1RA concentration actually reflects increased postoperative IL-1 activity. IL-1RA, IL-10, and MCP-1 were shown to play an important role in the recovery of monocyte function after pediatric cardiac surgery [34]. There have been studies on the pathophysiological role of IL-1RA in experimental pulmonary hypertension [35–37], but there are no studies in pediatric pulmonary hypertension, nor are there studies in the particular setting of perioperative pulmonary hypertension. Our data seem to indicate that both proinflammatory (IL-6) and anti-inflammatory responses (IL-1RA, G-CSF, and IL-21) were more prominent in patients with heightened postoperative pulmonary artery pressure. We speculate that the anti-inflammatory response, albeit evident, was not sufficiently intense to antagonize the effects of proinflammatory stimuli on pulmonary vascular tone in these individuals.

SCAPEs occurred in 27.5% of patients. This relatively high prevalence was due to the inclusion of events that would probably not be classified as pulmonary hypertensive crises based on contemporary definitions but contributed to the need for prolonged mechanical ventilation. Of the variables tested for correlation with the occurrence of SCAPEs, immediate postoperative hemodynamics and serum MIF 4 hours post-CPB reached statistical significance in univariate and multivariate analyses. Higher initial levels of pulmonary arterial pressure (i.e., PAP/SAP_IPO_ > 0.40) may have rendered some patients more susceptible to instabilities triggered by factors known to affect pulmonary vascular tone postoperatively, such as alveolar hypoxia, changes in pH, and right ventricular dysfunction. MIF is a noncanonical ligand of chemokine receptors known to play a central role in innate immunity [38] and a number of biological processes involved in vascular remodeling, including pulmonary vascular remodeling [39]. It binds to the receptors CXCR2, CXCR4, and CXCR2/CD74, leading to the activation of signaling pathways that results in vascular smooth muscle cell proliferation and inhibition of endothelial cell apoptosis, recruitment of peripheral blood mononuclear cells, and endothelial cell transition towards a proinflammatory phenotype [40–42]. Previously, we found associations of serum MIF with pulmonary arterial hypertrophy, pulmonary vascular resistance, and response to inhaled nitric oxide in pediatric subjects with congenital cardiac communications [43]. Of potential interest in terms of acute changes in pulmonary vascular tone, MIF was shown to enhance pulmonary vasoconstriction in response to hypoxia and potentiate constriction preevoked by agonists in isolated pulmonary artery rings [44].

## 5. Conclusion

Based on our findings, we conclude that pulmonary hemodynamic patterns may be identified early after surgery in patients with congenital cardiac communications. A relationship was demonstrated between postoperative systemic inflammatory response and the behavior of the pulmonary circulation. Early postoperative elevation of pulmonary arterial pressure was associated with relevant cardiopulmonary events, which in turn accounted for prolonged mechanical ventilation. Six markers of inflammation present in circulation were directly or indirectly associated with the occurrence of postoperative events. The relationships that we report between hemodynamics, inflammation, and outcome may have implications in terms of treatment planning and prevention of complications in this population.

## Figures and Tables

**Table 1:** Preoperative and intraoperative data of 40 patients.

Age (months)	11 (8–17)
Sex (M : F)	11 : 29
Weight (kg)	6.42 (5.72–7.91)
Height (cm)	69 (63–76)
Down syndrome, *n* (%)	25 (62.5)
Peripheral oxygen saturation (%)	96 (93–98)
*Cardiac anomaly*	
Ventricular septal defect, *n* (%)^∗^	24 (60.0)
Atrioventricular septal defect, *n* (%)^†^	16 (40.0)
*Echocardiographic parameters*	
Systolic pulmonary arterial pressure (mmHg)	75 (52–86)
Mean pulmonary arterial pressure (mmHg)	48 (34–54)
Pulmonary/systemic blood flow ratio	2.30 (1.80–3.20)
Velocity-time integral of blood flow in pulmonary veins (cm)^ǁ^	21.5 (20.1–25.0)
TAPSE (mm)^‡^	15.0 (13.0–17.8)
TAPSE, *Z*-score	0.50 (-1.60–1.93)
*Intraoperative parameters*	
Cardiopulmonary bypass (CPB) duration (min)	128 (90–152)
Mean pulmonary arterial pressure	
Before CPB (mmHg)	32 (26–36)
After CPB (mmHg)	22 (18–26)^§^
Mean systemic arterial pressure	
Before CPB (mmHg)	46 (40–54)
After CPB (mmHg)	53 (49–59)^§^
Pulmonary/systemic mean arterial pressure ratio	
Before CPB (mmHg)	0.73 (0.56–0.86)
After CPB (mmHg)	0.40 (0.33–0.48)^§^
Modified ultrafiltration volume (mL)	60.5 (41.3–70.6)

Numeric variables are presented as median with interquartile range. ^∗^Singly or in association with atrial septal defect and/or patent ductus arteriosus. ^†^Singly or in association with patent ductus arteriosus. ^ǁ^Values < 20 cm are generally associated with heightened pulmonary vascular resistance in pediatric patients with unrestrictive cardiac communications [14, 15]. ^‡^Tricuspid annular plane systolic excursion. Normally, values increase with increasing age in the pediatric population. A TAPSE of ≥15.5 is considered normal at the age of 1 year, as are values ≥ 16.5 by the age of 2 years [16]. ^§^*p* < 0.001 versus pre-CPB levels, Wilcoxon test.

**Table 2:** Preoperative and intraoperative data in hemodynamic groups.

	PAP/SAP_IPO_ ≤ 0.40 (*n* = 22)	PAP/SAP_IPO_ > 0.40 (*n* = 18)	*p* value
Age (months)	12 (8–20)	11 (8–15)	0.874
Sex (M : F)	6 : 16	5 : 13	0.999^∗^
Down syndrome, *n* (%)	11 (50)	14 (78)	0.140^†^
Peripheral oxygen saturation (%)	98 (96–99)	95 (92–96)	<0.001
Atrioventricular septal defect: ventricular septal defect	5 : 17	11 : 7	0.032^†^
Pulmonary/systemic blood flow ratio	2.45 (1.80–3.33)	2.15 (1.68–2.98)	0.360
Precardiopulmonary bypass (CPB) mean pulmonary arterial pressure (mmHg)	31 (23–35)	35 (29–39)	0.146
Pre-CPB pulmonary/systemic mean arterial pressure ratio	0.65 (0.50–0.80)	0.77 (0.66–0.87)	0.091
CPB duration (min.)	107 (86–135)	146 (121–159)	0.007

Numeric variables are presented as median with interquartile range. Differences were analyzed using the general linear model after Box-Cox transformation of dependent variables. ^∗^Fisher's exact test. ^†^Chi-square test.

**Table 3:** Preoperative and postoperative hematological parameters.

	Before surgery	Post-CPB			*p* value^∗^
		4 h	24 h	48 h	
White blood cell count (K/*μ*L)	11.04 (7.14-12.21)	14.06 (11.02-17.66)^†^	14.17 (11.86-18.09)^†^	14.75 (12.13-20.19)^†^	<0.001
Lymphocytes (K/*μ*L)	4.66 (3.66-7.76)	1.70 (1.02-2.41)^†^	1.87 (1.22-2.23)^†^	2.37 (1.51-2.98)^†^	<0.001
Neutrophils (K/*μ*L)	3.33 (2.00-4.91)	10.59 (8.28-13.86)^†^	10.64 (8.35-13.66)^†^	10.97 (7.61-15.29)^†^	<0.001
Neutrophil to lymphocyte ratio	0.57 (0.35-1.05)	6.83 (4.06-10.88)^†^	5.92 (4.28-9.25)^†^	5.07 (2.94-9.13)^†^	<0.001
Monocytes (K/*μ*L)	0.71 (0.50-1.04)	1.03 (0.73-1.48)^†^	1.47 (0.86-1.93)^†^	1.34 (0.85-1.81)^†^	<0.001
Platelets (K/*μ*L)	329 (272-390)	141 (118-170)^†^	136 (110-164)^†^	138 (107-178)^†^	<0.001
Mean platelet volume (fL)	9.50 (9.00-10.00)	9.40 (8.80-9.90)	10.05 (9.45-10.40)^‡^	10.30 (9.75-10.70)^‡^	<0.001
C-reactive protein (mg/L)	2.07 (1.17-4.57)	2.88 (1.78-3.76)	69.50 (53.80-81.80)^†^	136.19 (108.24-201.06)^†^	<0.001

Variables are presented as median with interquartile range. ^∗^Friedman test. † and ‡, respectively, *p* < 0.001 and *p* < 0.01 versus baseline (*post hoc* multiple comparisons). CPB: cardiopulmonary bypass.

**Table 4:** Baseline levels of inflammatory proteins.

	Controls (*n* = 37)	Patients (*n* = 40)	*p* value		Controls (*n* = 37)	Patients (*n* = 40)	*p* value
C5/C5a (upi)	1392 (804-3756)	2442 (1027-3980)	0.209	IL-13 (upi)	509 (294-1090)	654 (311-1256)	0.492
CD40L (upi)	4353 (2670-7079)	5233 (2871-7770)	0.677	IL-16 (upi)	521 (276-1116)	568 (262-1240)	0.749
G-CSF (upi)	347 (198-603)	560 (245-867)	0.120	IL-17 (upi)	257 (146-450)	300 (190-556)	0.204
GM-CSF (upi)	293 (146-356)	343 (168-492)	0.450	IL-17E (upi)	344 (183-565)	407 (224-786)	0.173
GRO*α* (upi)	1712 (1315-2578)	1770 (1204-2866)	0.824	IL-27 (upi)	221 (134-411)	304 (173-422)	0.401
I-309 (upi)	636 (318-802)	674 (255-1210)	0.301	IL-32*α* (upi)	470 (220-980)	447 (257-990)	0.993
ICAM-1 (upi)	38777 (30494-49330)	39756 (29716-48504)	0.847	IP-10 (upi)	646 (246-1185)	827 (432-1941)	0.052
IFN-*γ* (upi)	269 (168-389)	329 (190-438)	0.434	I-TAC (upi)	522 (243-989)	706 (309-1024)	0.459
IL-1*α* (upi)	363 (169-589)	423 (208-712)	0.407	MCP-1 (upi)	345 (261-637)	491 (238-876)	0.319
IL-1*β* (upi)	191 (100-386)	185 (116-515)	0.766	MIF (upi)	6339 (5432-6838)	5677 (4635-7169)	0.444
IL-1RA (upi)	1407 (759-2276)	1571 (605-2493)	0.903	MIP-1*α*/*β* (upi)	409 (232-580)	601 (249-914)	0.093
IL-2 (upi)	268 (119-415)	324 (121-511)	0.378	Serpin E1 (upi)	44804 (40018-51686)	46656 (35898-61188)	0.670
IL-4 (upi)	276 (179-444)	344 (208-510)	0.333	RANTES (upi)	58061 (47531-65624)	66140 (53982-76405)	0.012
IL-5 (upi)	98 (56-198)	94 (76-226)	0.601	SDF-1 (upi)	3892 (3250-5036)	4251 (3323-5967)	0.419
IL-6 (upi)	560 (260-983)	301 (192-718)	0.197	TNF-*α* (upi)	260 (144-473)	342 (151-629)	0.508
IL-8 (upi)	202 (138-396)	282 (155-509)	0.272	sTREM-1 (upi)	264 (151-543)	381 (235-595)	0.312
IL-10 (upi)	276 (132-399)	277 (197-490)	0.809	IL-18 (upi)	874 (500-1718)	951 (527-1602)	0.909
IL-12p70 (upi)	243 (119-465)	261 (142-443)	0.612	IL-21 (upi)	401 (185-612)	379 (253-789)	0.512

Data are presented as median with interquartile range. Levels of inflammatory markers are given in units of pixel intensity (upi, chemiluminescence). Differences were analyzed using the general linear model after Box-Cox transformation of dependent variables.

**Table 5:** Inflammatory markers in groups defined according to immediate postoperative pulmonary hemodynamics.

	PAP/SAP_IPO_ ≤ 0.40 (*n* = 22)			PAP/SAP_IPO_ > 0.40 (*n* = 18)			*p* value	
	Before surgery	Post-CPB		Before surgery	Post-CPB		Within subjects	Between groups
		4 h	24 h		4 h	24 h		
CD40L (upi)	4310 (2740-8359)	1898 (1178-2993)	2307 (1585-4034)	5916 (3162-6983)	2142 (1263-5321)	2760 (1315-4373)	<0.001	0.873
G-CSF (upi)	391 (154-800)	814 (360-1397)	401 (247-619)	689 (294-923)	1234 (766-1949)	679 (343-1597)	<0.001	0.040
GRO*α* (upi)	1588 (1269-2980)	1191 (684-1353)	741 (443-1476)	1796 (1189-2947)	1594 (683-2828)	1210 (646-1670)	<0.001	0.292
IL-1RA (upi)	1210 (518-2249)	13140 (9524-18184)	4662 (1770-6574)	1925 (1070-2633)	18853 (14058-21786)	7036 (4654-12461)	<0.001	0.020
IL-6 (upi)	281 (141-637)	826 (368-1215)	601 (330-1071)	477 (225-1210)	2056 (1010-2839)	961 (450-2181)	<0.001	0.003
IL-8 (upi)	279 (146-403)	292 (169-621)	227 (144-448)	317 (155-655)	511 (267-864)	269 (189-610)	0.014	0.167
IL-16 (upi)	564 (261-1083)	1241 (1001-2715)	908 (349-1218)	578 (448-1518)	2768 (1529-3923)	1133 (635-2878)	<0.001	0.155
IP-10 (upi)	904 (410-1808)	1466 (637-2723)	659 (166-1122)	824 (623-2033)	3186 (1015-4080)	702 (186-2109)	<0.001	0.305
MCP-1 (upi)	381 (230-786)	447 (323-830)	364 (254-631)	560 (246-962)	881 (610-1269)	508 (241-1100)	0.002	0.178
MIF (upi)	5514 (4113-7055)	7117 (6604-9722)	5587 (3662-8508)	6078 (5192-7330)	9010 (7209-11057)	6753 (5465-8794)	<0.001	0.299
Serpin E1 (upi)	44650 (35485-57863)	37559 (30688-46958)	34719 (27139-40310)	52910 (40782-62429)	44831 (32830-53588)	40841 (35076-51601)	<0.001	0.086
RANTES (upi)	64525 (50627-81348)	50828 (39911-78459)	46564 (36403-66291)	68037 (59861-74569)	51934 (42454-68576)	48699 (33868-60721)	<0.001	0.956
SDF-1 (upi)	4123 (3615-6061)	3807 (2486-7486)	2668 (2098-4702)	4695 (3031-5795)	4797 (4031-7014)	3566 (2980-4947)	0.002	0.734
IL-21 (upi)	338 (152-589)	349 (257-558)	321 (211-585)	658 (344-949)	562 (353-912)	479 (278-834)	0.181	0.047
MPV (fL)	9.40 (8.65-9.80)	9.20 (8.65-9.85)	9.80 (9.20-10.30)	10.00 (9.30-10.85)	9.40 (9.05-10.00)	10.30 (9.75-10.85)	<0.001	0.018
NLR	0.54 (0.38-0.81)	6.83 (4.23-9.41)	5.14 (3.54-9.17)	0.77 (0.43-1.40)	6.50 (3.85-16.72)	7.09 (4.82-9.28)	<0.001	0.148
CRP (mg/L)	2.62 (1.27-4.99)	2.05 (1.56-3.20)	61.99 (51.09-78.33)	1.54 (0.90-3.39)	3.05 (2.01-4.35)	69.12 (55.62-82.65)	<0.001	0.942

Shown are the markers for which significant differences (within subjects and/or between groups) were observed. Results are presented as median with interquartile range. Levels of proteins obtained by chemiluminescence are given in units of pixel intensity (upi). Data were analyzed after Box-Cox transformation using the general linear model for repeated measures. PAP/SAP_IPO_: pulmonary/systemic mean arterial pressure ratio, mean of first 4 values (2-hour intervals) obtained in the postoperative care unit; CPB: cardiopulmonary bypass; CRP: C-reactive protein; MPV: mean platelet volume; NLR: neutrophil to lymphocyte ratio.

**Table 6:** Variables associated with significant postoperative cardiopulmonary events (SCAPEs).

	SCAPEs		*p* value		
	No (*n* = 29)	Yes (*n* = 11)	Univariate analysis	Multivariate analysis 1	Multivariate analysis 2
PAP/SAP_IPO_ > 0.40, *n* (%)	10 (35)	8 (73)	0.038	0.046	0.047
CD40L at baseline (upi)	4348 (2871-6863)	6163 (3642-11641)	0.078	0.291	—
IL-6 at baseline (upi)	281 (180-637)	499 (179-1267)	0.079	0.406	—
IL-8 at baseline (upi)	188 (119-351)	403 (283-618)	0.089	0.767	—
IL-16 on day 0 (upi)	1241 (956-2767)	2140 (1265-5703)	0.048	0.859	—
IL-1RA on day 0 (upi)	14181 (9916-19524)	20302 (14144-22796)	0.078	0.446	—
MIF on day 0 (upi)	7221 (6660-9356)	10851 (7224-14947)	0.021	0.059	0.023
RANTES on day 0 (upi)	49528 (41541-59176)	69437 (40039-95821)	0.080	0.451	—
SDF-1 on day 0 (upi)	4131 (2477-6277)	6584 (3931-10135)	0.075	0.878	—
NLR on day 0	7.58 (4.42-11.71)	3.79 (2.24-6.82)	0.043	0.105	—
CRP on day 2 (mg/L)	118.89 (95.70-193.51)	163.43 (136.59-213.60)	0.077	0.419	—

Variables were tested using logistic regression. Shown are those with *p* < 0.10 in univariate analysis. In multivariate analysis 1, all variables were included in the model. In multivariate analysis 2, the forward LR procedure was used for variable selection. PAP/SAP_IPO_: pulmonary/systemic mean arterial pressure ratio, mean of first 4 values obtained at the postoperative care unit; CRP: C-reactive protein; NLR: neutrophil to lymphocyte ratio; baseline: day 0 and day 2, respectively, preoperative determination, 4 hours after cardiopulmonary bypass and 2 days after surgery.

## Data Availability

The data used to support the conclusion of the present study correspond to the project FAPESP #2015/21587-5 and are available from the corresponding author upon request.
